# Oesophageal cancer: exploring controversies overview of experts’ opinions of Austria, Germany, France, Netherlands and Switzerland

**DOI:** 10.1186/s13014-015-0418-4

**Published:** 2015-05-21

**Authors:** Paul Martin Putora, Laurent Bedenne, Wilfried Budach, Wolfgang Eisterer, Ate Van Der Gaast, Robert Jäger, J. Jan B. Van Lanschot, Christophe Mariette, Annelies Schnider, Michael Stahl, Thomas Ruhstaller

**Affiliations:** Department of Radiation Oncology, Kantonsspital St. Gallen, 9007 St. Gallen, Switzerland; University Hospital Le Bocage, Dijon, France Federation Francophone de Cancérologie Digestive (FFCD), Dijon, France; Department of Radiation Oncology, Heinrich Heine University of Düsseldorf, German Oesophageal Study Group (GOSG), Düsseldorf, Germany; Department of Internal Medicine V, Innsbruck Medical University, West-Austrian Study Group (AGMT), Innsbruck, Austria; Department of Medical Oncology, Erasmus University Medical Center, ChemoRadiotherapy for Oesophageal cancer versus Surgery alone Study Group (CROSS), Rotterdam, The Netherlands; Department of Radiation Oncology, Innsbruck Medical University, West-Austrian Study Group (AGMT), Innsbruck, Austria; Department of Surgery, Erasmus University Medical Center, ChemoRadiotherapy for Oesophageal cancer versus Surgery alone Study Group (CROSS), Rotterdam, The Netherlands; Department of Digestive and Oncological Surgery, University Hospital Lille, Federation Francophone de Cancérologie Digestive (FFCD), Lille, France; Department of Surgery, City-Hospital Triemli, Swiss Group for Clinical Cancer Research (SAKK), Zurich, Switzerland; Kliniken Essen-Mitte, Klinik für Internistische Onkologie und Hämatologie, German Oesophageal Study Group (GOSG), Essen, Germany; Department of Haematology and Oncology, Swiss Group for Clinical Cancer Research (SAKK), Kantonsspital St. Gallen, St. Gallen, Switzerland

**Keywords:** Oesophageal cancer, Oesophagus, Treatment, Consensus, Controversy, European, Surgery, Radiotherapy, Chemotherapy

## Abstract

**Background:**

Oesophageal carcinoma is a rare disease with often dismal prognosis. Despite multiple trials addressing specific issues, currently, many questions in management remain unanswered. This work aimed to specifically address areas in the management of oesophageal cancer where high level evidence is not available, performing trials is very demanding and for many questions high-level evidence will not be available in the forseeable future.

**Methods:**

Two experts of each national, oesophageal cancer research group from Austria, France, Germany, the Netherlands and Switzerland were asked to provide statements to controversial issues. After an initial survey, further questions were formulated and answered by all experts. The answers were then discussed and qualitatively analysed for consensus and controversy.

**Results:**

Topics such as indications for PET-CT, reasons for induction chemotherapy, radiotherapy dose, the choice of definitive chemo-radiotherapy versus surgery in squamous cell cancer, the role of radiotherapy in adenocarcinoma and selected surgical issues were identified as topics of interest and discussed.

**Conclusion:**

Areas of significant controversy exist in the management of oesophageal cancer, mostly due to high-level evidence. This is not expected to change in the upcoming years.

**Electronic supplementary material:**

The online version of this article (doi:10.1186/s13014-015-0418-4) contains supplementary material, which is available to authorized users.

## Introduction

Oesophageal carcinoma is a relatively rare disease with a still dismal prognosis despite having seen some improvement in management over the last decade mainly reflecting better patient selection, more effective neo-adjuvant therapy and improved perioperative management. There remain many unresolved issues and existing controversies. Published clinical trials often provide insufficient answers for individual patients; too many variables influence the treatment decision such as different histologies implying different clinical biology, the location of the tumour influencing the preferred technique of surgery and radiotherapy or significant co-morbidities of the patient. With the relative rarity of this disease and the requirement for intensive teamwork in diagnostics and therapeutics, large multi-centre studies are difficult to perform and are often underpowered and their results may be challenging to translate into clinical practice.

Clinicians must make decisions despite gaps in evidence and many issues will not be solved within clinical trials over the next decade. In an attempt to provide guidance in these situations [1], various consensus methodologies have been established [2, 3]. Consensus meetings typically gather specialists in a field, facilitate exchange of opinions, and usually result in a minimal consensus statement. In 2012, under the auspices of the EORTC, a consensus meeting was held in St. Gallen, Switzerland with the aim to produce a consensus statement on “the primary therapy of gastric, gastro-oesophageal and oesophageal cancer-differential treatment strategies for subtypes of early gastro-oesophageal cancer” [4]. Allthough this statement did provide minimal consensus on various issues, many questions remained unanswered.

The aim of this work is focussing particularly on controversial issues in the therapy of locally advanced oesophageal cancer in contrast to the published minimal consensus statement. Prominent representatives of the national cancer research groups (NCRG) dealing with oesophageal cancer from Austria, France, Germany, the Netherlands and Switzerland discussed these issues and explained their views and opinions and provided practical advice. The aim of this work is to demonstrate the spectrum of opinions on various critical issues and provide practical approaches where evidence is not available.

We hope our work will support clinicians in their daily practice and research groups in determining areas for future studies.

## Methods

After the first EORTC consensus meeting on treatment standards in St. Gallen 2012 the idea of a more detailed discussion of the unresolved controversies came up. Leaders of several European cancer research groups were asked to support this initiative. Representatives of the French Group FFCD (“Federation Francophone de Cancérologie Digestive”), the Swiss Group for Clinical Cancer Research (SAKK), the German Oesophageal Study Group, the Austrian Group (West-Austrian Study group) and the Dutch Group (CROSS study group) agreed to participate; whereas the Upper GI Clinical Studies Group of the National Cancer Research Institute UK was invited but decided not to participate. From each participating group two representatives of different therapeutic disciplines were determined, in summary: 3 surgeons, 5 medical oncologists and 2 radiation oncologists, additionally one Swiss radiation oncologist (PMP), the co-initiator, was part of the study team.

Based on the consensus statement on oesophageal cancer published in 2012 a catalogue of controversial issues and questions was generated. An initial draft was then sent to the representatives of each group with the request to answer the proposed questions of controversy based on the opinions and standards within their national research group.

After receiving the first answers to the survey, several questions were further specified based on obtained input. Participants were once again invited to add further questions they considered of interest. After including the new questions, every participant received the survey (Additional file 1) again, this time including the responses from all participants. Following this, a third round was initiated to provide room for clarifications, questions and answers and also discussions between the different groups.

After the final list of answers was collected, questions considered of little relevance or those without general interest were discarded. The remaining statements were summarised by topics. Based on these and a further discussion among all, the representatives were asked to provide a statement of support or comment on all issues.

This manuscript intentionally also addresses topics where no consensus was reached in order to highlight the controversies; explanations of different opinions were included.

The expressed opinions from the prominent representatives of the research groups were not distributed and compared in their national groups; However, the leaders have been involved in the design and execution of several study protocols in this field and also have insight into ongoing trials within their research groups.

## Results

### PET-CT-SCAN

#### Should PET-CTs be used for staging?

All NCRGs consider 18-FDG-PET-CT scans a useful tool for the detection of additional metastases, particularly for locally advanced tumours. Experts from France did not recommend routine use of PET-CTs, especially when EUS and CT scans are performed in expert centres. Although a relatively high proportion of ACs might not show FDG avidity, the recommendations were independent of histology. The diagnosis “oesophageal cancer” is sufficient for reimbursement in Switzerland and France and soon in the Netherlands. Though not systematically re-imbursed, most centres in Austria and Germany make use of PET-CTs for patients with oesophageal carcinomas. However, in Germany and the Netherlands the missing reimbursement is a limiting factor. The new guidelines in the Netherlands will recommend PET-CTs for pre-treatment staging as well as for re-staging after neoadjuvant radiochemotherapy.

#### PET-CTs for radiation therapy treatment planning?

In addition to staging, PET-CT is being incorporated in radiotherapy planning in all NCRGs, it appears to be especially relevant for lymph nodes marginally outside the standard radiotherapy treatment volumes. In Austria the PET-CT is also used to plan a radiotherapy boost. In the Netherlands, radiotherapy volumes are only adapted based on lymph nodes outside the planned field after invasive verification (EUS-FNA/FNB).

#### How do you deal with PET-positive hilar lymph nodes?

Major variations were observed for the management of PET-positive hilar lymph nodes. The different opinions reached from considering FDG-positive hilar lymph nodes of normal size not pathologic to considering them metastatic disease. The patterns of practice are depicted in Table 1.

**Table 1:** Management of PET-positive hilar lymph nodes

France	Positive hilar lymph nodes of normal size are not considered pathologic. Since they are generally associated with other lymph node sites, a neoadjuvant chemotherapy is provided. if right-sided location: removed at time of EC surgery; if left-sided location: cytological verification required and if positive: included in the radiation field
Netherlands	FNA/Biopsy: if malignant, usually considered to be distant metastases
Germany	The radiation portals are modified to include the PET-positive nodes
Austria	Treated if tumour location is in the middle third of the oesophagus and nodes are enlarged on CT
Switzerland	If positive lymph nodes are small (<1.5 cm) and patient is a smoker, no further investigations recommended. If the lymph nodes are larger and without other plausible cause, FNA is performed. If positive, included in radiotherapy field.

### Induction chemotherapy

Although level 1 evidence for induction chemotherapy before CRT is missing, most NCRGs are using induction chemotherapy in their study protocols. Several reasons may justify induction chemotherapy (see Table 2). Histology plays no role in this decision, it is also independent of further therapy, CRT followed by surgery or definitive CRT, respectively. In the Netherlands and in France routine use of induction chemotherapy is not considered. Most centres consider two – three cycles adequate, not delaying surgery excessively. The reasons to consider induction chemotherapy are listed in the Table 2, however this approach remains highly controversial.

**Table 2:** Reasons to consider induction chemotherapy

• rapid start of treatment	• fast improvement of dysphagia, feeding tubes can nearly always be avoided
• test of patient compliance also to consider further therapy decisions	• increase of compliance of the following CRT because of better nutritional status and less dysphagia
• *in vivo* testing of chemotherapy before the start of radiotherapy	• more and early systemic treatment for possible metastatic disease (most patients still die of systemic disease)

### SCC of esophagus

#### When should definitive chemo-radiation be preferred over a surgical approach?

According to the consensus conference all countries except the Netherlands and France prefer definitive CRT for tumours located in the upper thoracic oesophagus. They do not start to evaluate surgery unless the proximal edge of the tumour has clearly 4–5 cm distance from the entrance of the oesophagus (thoracic inlet). In the Netherlands and in France only cervical oesophageal cancer is treated with CRT only, surgery is evaluated for all intra-thoracic tumours.

The following reasons were named by all parties as leading to a preference for definitive CRT over surgery independent of the location: patient preference, co-morbidities, high biological age and high perioperative risk. Additionally tumours infiltrating the laryngeal nerve, the upper oesophageal sphincter, thoracic aorta or the tracheo-bronchial tree are also unsuitable for operation. For tumours invading the tracheo-bronchial tree, starting treatment with neoadjuvant chemotherapy alone to prevent fistula was recommended. A large tumour mass and a tumour extending >10 cm could also be reason to prefer definitive CRT.

#### How to proceed after cCR after neodjuvant radiochemotherapy?

In Germany and in France omitting the operation would be considered a reasonable alternative outside of clinical trials and an option after discussion with the patient.

In all other countries this approach would explicitly not be considered a standard and the operation would be recommended in spite of cCR after neoadjuvant therapy.

#### Which dose of radiotherapy is justified for neoadjuvant or definitive CRT?

There was controversy about the correct dose of radiotherapy, however all NCRGs agreed that when individual patient characteristics were considered (poor performance status, comorbidities, age..) any dose in the mentioned range would be considered acceptable. There was no clear recommendation on the use of a radiotherapy boost apart from Austria. A dose that is considered feasible by all was 45 Gy in the neoadjuvant setting and ranging from 50. 4 Gy to 60 Gy for definitive radio-chemotherapy with 1.8 Gy to 2.0 Gy per fraction 5x per week (Table 3). Radiotherapy dose escalation (64.8 Gy vs. 50.4 Gy) within definitive radio-chemotherapy was investigated in a phase III RTOG 9405/INT 0123 trial [5], however due to a high number of early deaths in the high dose arm, the results are perceived as controversial. It has to be realised that the more often performed rescue surgery after definitive CRT and local relapse is associated with a higher risk of postoperative morbidity if a radiotherapy dose of more than 50 Gy was applied.

**Table 3:** Radiation doses used for neoadjuvant as well as definitive radiochemotherapy

Neoadjuvant:	Definitive:
45 Gy (France, Austria, Switzerland)	50.4 Gy, without boost (France, Netherlands)
	At least 60 Gy (Germany)
41.4 Gy (Netherlands)	50.4 Gy to 59.4 Gy +/− brachytherapy boost (Austria)
40–50 Gy (Germany)	In Switzerland very heterogenous opinions from 50.4 Gy to 59.4 Gy (without boost or brachytherapy)

#### How should patients be restaged after neoadjuvant CRT?

EUS after neoadjuvant CRT was not considered relevant. When patients are restaged to prepare for surgery, a CT scan is performed by all NCRGs. In France, Germany and Austria endoscopy is repeated, while PET-CT for re-staging is not performed routinely outside of studies after neoadjuvant CRT. In the new Dutch guidelines PET-CTs for restaging after CRT are recommended.

When omitting surgery is considered in case of cCR several biopsies in the tumour area were considered mandatory and non-invasive imaging alone insufficient.

### Esophago-gastric junction adenocarcinoma (Siewert I and II)

The treatment of choice is generally neoadjuvant CRT. In France, as well as in Germany, neoadjuvant / perioperative chemotherapy is standard for adenocarcinoma Siewert type II, except R0 resection is not deemed achievable. It is worth mentioning that the differentiation between Siewert types may be difficult in some cases despite radiographic or even endoscopic data. All agreed that the preference to include radiotherapy increased with extension into the oesophagus. Neoadjuvant chemotherapy alone was the second best option, but should trimodality therapy be considered too risky (e.g. comorbidities, risk of surgery) then omitting radiotherapy instead of surgery would be preferred. This is obviously in contrast to SCC of the oesophagus. Definitive chemoradiotherapy was considered an option only if the other two previously mentioned treatments were not possible (“third best option”). Most agreed that coeliac lymph-nodes would be included in the radiation fields, in the Netherlands these would be omitted if clinically bland.

### Surgical issues

#### Preferred oesophagectomy technique?

Transthoracic en-bloc esophagectomy with two-field lymphadenectomy in a one-stage procedure was preferred by all. The anastomosis would be preferred to be intra-thoracic at the upper part of the thorax (France, Austria) or cervical (Netherlands), with no consensus among the surgeons in Germany and Switzerland. Cervical anastomosis was preferred for those with upper-third cancers (in order to reach microscopically negative proximal margins; or because of better prognosis in case of leakage). Upper intrathoracic anastomosis was preferred over cervical due to lower percentage of leakages and lower stricture rates.

#### The use of minimally invasive oesophagectomy (MIO)

Despite limited follow-up and high requirements in expertise, in most centres MIO was considered an option, however the implementation was highly variable among the centres responding (see Table 4).

**Table 4:** Implementation of minimally invasive oesophagectomy

Austria	No general consensus about technique, but in some specialised centres either laparoscopy or thoracoscopy is performed in the surgical treatment of oesophageal cancer. Most of these use hybrid techniques, i.e. one part minimal invasive, the other part open.
France	Hybrid minimally invasive is routine in experienced centres with laparoscopic gastric mobilisation and open thoracotomy. The aim is to decrease the rate of major postoperative pulmonary complications. Thoracoscopic oesophagectomy is reserved for high-grade dysplasia or early tumour, because it is considered that large en bloc resection for locally advanced cannot be safely done in routine
Germany	Minimal invasive surgery for the thoracic part is meanwhile the preferred in the participant’s centre (Essen), but there is no consensus in the group.
Netherlands	MIO is mainly applied for early cases. Main advantage: less pulmonary complications. After the Dutch RCT MIO is gradually also accepted for more advanced cases
Switzerland	Minimally invasive surgery is a good option for the thoracic part, especially when a cervical anastomosis is done. Others prefer a laparascopic gastric mobilisation followed by open thoracotomy and a thoracic anastomosis. There is no consensus in the group.

#### What are the indications for a transhiatal resection in oesophago-gastric junction carcinoma (Siewert type I or II)?

Most agreed on transhiatal resection for Siewert II Tumour (Austria, Netherlands, Switzerland, Germany) and transthoracal approach for Siewert I tumours. Thoracotomy would be preferred for both. In France, the transhiatal approach would be reserved for patients not tolerating thoracotomy.

#### Which surgical technique is preferred for the special case of a SCC at the oesophago-gastric junction?

Transthoracic resection in all squamous cancers was preferred in France, the Netherlands and Austria independent of the location. Transmediastinal esophagectomy would be an alternative in combination with bilateral mediastinal “en bloc” lymphadenectomy (para-aortic nodes, para-oesophageal nodes, pulmonary left and right hilar nodes including those of the tracheal bifurcation) as a two field lymphadenodectomy in Switzerland. This would also be considered in Germany after CRT.

## Discussion

Our analysis identified consensus as well as controversies in the management of oesophageal cancer, particularly where evidence is missing. These results were influenced by the selection of groups and their representatives. Nonetheless, the groups represent a significant share of European clinical research in gastrointestinal cancer.

The interdisciplinary and international nature of this collaboration has proven valuable. Interestingly, controversies based on modality thinking did not appear (e.g. surgery vs. radiotherapy) and generally a high acceptance of discrepancy was present.

Based on the results of this analysis, the authors conclude that several areas of high controversy are amenable to future trials, some of which are either ongoing or in advanced stages of planning (Table 5).

**Table 5:** A selection of prospective trials in localised oesophageal cancer in the participating countries

Group/Name of the trial	Background Objectives	Endpoints Design	Sample size	(planned) date of trial initiation
SAKK 75/08 (Swiss, German, Austrian and French centers) NCT01107639	Multimodal therapy with and without cetuximab, locally advanced esophageal carcinoma.	Ph-lll: PFS	300	Accrual fulfilled, results expected Q15
FFCD/FRENCH (ESOSTRATE trial)	Operable oesophageal cancer with clinical complete response after neoadjuvant chemoradiation randomised between systematic surgery vs surveillance with selective salvage surgery in case of operable recurrence	Ph-ll: Percentage of patients alive at 1 year >70 % in the surveillance arm Ph-lll: DFS	Pts randomised with cCR: Ph-ll: 114 Ph-lll: 260	1Q 2015
Open Versus Laparoscopically-assisted Esophagectomy for Cancer (MIRO) FREGAT NCT00937456	Hybrid MIO vs open oesophagectomy	Ph. III: overall morbidity, DFS, OS, QoL, economical interest of the surgical technique through a hospital point of view	200	Closed to recruitement
MAGIC vs. CROSS Upper GI. ICORG 10–14, V3 (NEOAEGIS) NCT01726452	Phase III trial comparing neoadjuvant chemotherapy to chemoradiation in junctional adenocarcinomas (NEOAEGIS)	Ph. III: OS, clinical and pathological response rate, health-related QoL, tumour regression grade, node-positivity, post-operative pathology, DFS, time to treatment failure, toxicity, post-operative complications.	366	ongoing
PROTECT-01 trial FFCD-UNICANCER-FREGAT (NCT02359968)	Phase II/III randomised comparison of preoperative chemoradiation with paclitaxel-carboplatine or with fluorouracil-oxaliplatine-folinic acid (FOLFOX) for unresectable esophageal and junctional cancer	Ph II: complete surgical resection (R0) and severe postoperative morbidity Ph III: overall survival	Ph II: 96 Ph III 400	Q1 2015
FREGAT database fregat-database.org	French prospective national database collecting epidemiological, clinical, pathological, biological, HRQOL and social data on esophageal and gastric cancer	not applicable	15 000	On going
Art-Deco trial (NTR3532)	Dose escalation in irresectable T4- tumours: 50.4 Gy versus 61. Gy plus carboplatin/paclitaxel	Ph. III: local tumour control in the esophagus	2x 130 patients	Since 2012
Pre-SANO trial (NTR4834)	Accuracy of assessment of tumour response after CROSS	Prospective, non-randomised: correlation between cCR and pCR 12 weeks after end of CROSS	140	Since 2013
Robot-assisted Thoraco-laparoscopic Esophagectomy Versus Open Transthoracic Esophagectomy (ROBOT) NCT01544790	Evaluate the benefits, risks and costs of robot-assisted thoraco-laparoscopic esophagectomy as an alternative to open transthoracic esophagectomy as treatment for esophageal cancer.	Ph-lll	112	Since 2012
Feasibility Study of Chemoradiation, TRAstuzumab and Pertuzumab in Resectable HER2+ Esophageal Carcinoma (TRAP) NCT02120911	CROSS + Trastuzumab + Pertuzumab in HER positive tumours	Ph I/II: Safety and efficacy; % of patients completing trastuzumab and pertuzumab treatment	40	Since 2014
TOR trial NTR3060 RACE	High dose CRT followed by exploratory thoracotomy for cT4 tumours	The ability to achieve a radical (R0) resection.	30	2012
	Prospective randomised comparison of neoadjuvant radiochemotherapy versus chemotherapy in patients with resectable adenocarcinomas of the oesophago-gastric junction	Multicentre phase II/III trial with a 15 % improvement of 3-year DFS by neoadjuvant radio-chemotherapy compared to chemotherapy as primary endpoint	300 patients	2015

Although several controversial issues were identified, not all could be “solved”. However, in some situations certain ranges of what is considered “adequate” by the selected experts could be established and may be helpful in daily practice. Although these are not based on high level evidence, we believe that the structured discussion among experts can provide additional information for clinical decision-making beyond available evidence in a field where resources for studies are limited. A Summary of key points is provided.

## Conclusion - key points

- PET-CT for initial staging is considered to be a valuable tool for the detection of additional metastases and is also used for radiotherapy planning.
- Despite limited published evidence, induction chemotherapy is often being implemented. Improvement of dysphagia, quick start of therapy and control of systemic disease are the most prominent reasons for this strategy.
- 41.4–45 Gy are considered adequate in the neoadjuvant setting. The dose for definitive CRT is much more controversial, the opinions range from 50.4 Gy to over 60 Gy.
- After neoadjuvant CRT, restaging with CT scan is sufficient, some countries repeat endoscopy. PET-CT is not part of standard preoperative restaging so far.
- Most agreed on transhiatal resection for Siewert II tumours and transthoracal approach for Siewert I tumours (transthoracical en-bloc esophagectomy).
- Despite limited follow-up and high requirements in expertise, in most centres, minimally invasive surgery is considered an option, several studies are ongoing.
- Squamous cell cancer
  - ○ Most countries prefer definitive CRT for SCC located in the upper thoracic oesophagus
  - ○ Reasons preferring definitive CRT over surgery independent of the location: patient preference, co-morbidities, high biological age and high perioperative risk.
  - ○ When cCR is achieved after neoadjuvant therapy, omitting surgery could be an option when supported by patient preference. However, this option is not routinely recommended.
  - ○ In the presence of a large tumour mass and when the length of the tumour exceeds 10 cm a surgical approach should be considered with caution. T4b tumours should not be operated when radiochemotherapy is feasible.
- Adenocarcinoma
  - ○ In general neoadjuvant CRT is the treatment of choice for adenocarcinoma of the junction (Siewert I/II), the preference to include radiotherapy increased with extension into the oesophagus. Neoadjuvant chemotherapy alone is the second best option, if trimodality therapy is considered too risky (e.g. comorbidities, risk of surgery). Then omitting radiotherapy instead of surgery would be preferred.
  - ○ Several practical reasons like co-morbidities, poor performance status, etc. can lead to different treatment recommendations.

## Additional file

Additional file 1:
**Controversies: Treatment of oesophageal carcinoma Survey 1.0.**

## Abbreviations

18-FDG-PET-CT: Fluorine-18 Fluorodeoxyglucose positron emission tomography computed tomography
AC: Adenocarcinoma
AGMT: West-austrian study group
cCR: Clinical complete remission
CROSS: Chemo radiotherapy for oesophageal cancer versus surgery alone study group
CRT: Chemoradiotherapy
EORTC: European organisation for research and treatment of cancer
EUS: Endoscopic ultrasound
FFCD: Federation francophone de cancérologie digestive
FNA: Fine needle aspirate
FNB: Fine needle biopsy
GOSG: German oesophageal study group
Gy: Gray
MIO: Minimally invasive oesophagectomy
NCRG: National cancer research groups
RTOG: Radiation therapy oncology group
SAKK: Swiss group for clinical cancer research
SCC: Squamous cell cancer
FREGAT: For French Research in Esophageal and Gastric Tumours
