# Supplementary material for: Oesophageal cancer: exploring controversies overview of experts’ opinions of Austria, Germany, France, Netherlands and Switzerland
Source: Radiat Oncol. 2015 May 21;10:116. doi: 10.1186/s13014-015-0418-4 (PMC4461999; doi:10.1186/s13014-015-0418-4)
Supplement: Additional file 1: — Controversies: Treatment of oesophageal carcinoma Survey 1.0. [file 13014_2015_418_MOESM1_ESM.docx]

Controversies: Treatment of oesophageal carcinoma

Survey 1.0

| When filling out this survey feel free to copy-paste images,  tables or diagrams into this document.  Please return to ---  Thank you for your contribution,  --- |
| --- |

NAME OF RESPONDENT:

DATE:

**PET-CT FOR INITIAL STAGING**

**Reasons for and against recommending a PET-CT:**

- Useful for detection of additional metastases?
- Any differences between SCC and AC?
- Is reimbursement a factor in decision-making?
- What would change if reimbursement was no problem?

|  |
| --- |

Should PET-CT imaging be used for planning of radiotherapy target volumes?

|  |
| --- |

How do you deal with PET-positive hilar lymph nodes?

|  |
| --- |

**UPPER (THORACAL) ESOPHAGEAL CANCER**

*Standard therapy: definitive CRT is the treatment of choice, no role for surgery*

How do you define the term “upper oesophageal cancer”, where is the proximal and distal border? (Distance from incisors / anatomic landmark)

|  |
| --- |

From where do you start to evaluate for surgery? (25cm ab ore, below tracheal bifurcation, other?)

|  |
| --- |

Which radiotherapy dose? Which concomitant chemotherapy?

|  |
| --- |

**SCC OF ESOPHAGUS (MIDDLE&LOWER THIRD)**

*resectable stage and operable patients, standard therapy according to consensus: CRT followed by oesophagectomy*

What could be reasons not to go for surgery and to prefer definitive chemoradiation?

|  |
| --- |

Are there any stages in which you tend more to definitive CRT?

|  |
| --- |

Which dose of radiotherapy in neoadjuvant and definitive CRT? Do you use or recommend dose escalation of radiotherapy for definitive CRT with brachytherapy boost?

|  |
| --- |

**Achieving clinically complete remission after CRT**

- Could that be a reason to cancel initially planned surgery?

|  |
| --- |

- How and when to assess the clinical response to neoadjuvant treatment?

|  |
| --- |

- What gap of radiotherapy between stop of radiotherapy, restaging and restart of radiotherapy is acceptable? Are longer gaps compensated by higher dose?

|  |
| --- |

**ESOPHAGO-GASTRIC JUNCTION CARCINOMA**

*Adenocarcinoma, at typical localisation, Siewert l or ll, excluded Siewert lll*

What are the reasons to choose neoadjuvant CRT or CT only?

|  |
| --- |

Which lymphatic regions should be irradiated in primary radiotherapy?

|  |
| --- |

Could definitive CRT in some cases be an option? In which situations?

|  |
| --- |

**RADIOTHERAPY**

Which dose constraints for the lungs do you aim for primary treatment and neoadjuvant: e.g. V20 both lungs, mean lung dose, heart..

|  |
| --- |

When PET is not available, how is the tumour extent defined? (barium, CT, clips)

|  |
| --- |

Do you see any role for adjuvant brachytherapy after curative radiochemotherapy in clinical routine?

|  |
| --- |

**SURGERY**

**Surgery for SCC** *(oesophagectomy as standard)*

- Do you recommend resection of the below mentioned lymph nodes routinely:

| Lymph nodes: | routinely resected: | If YES, under which conditions: |
| --- | --- | --- |
| mediastinal |  |  |
| pulmonary |  |  |
| hilar |  |  |

- What is your preferred option for **oesophagectomy:**

Preferred localisation of anastomosis (why?), one-time or two-time surgery? Etc.

|  |
| --- |

- Is **mini-invasive surgery** an option or the preferred technique in your centre?

Mini-invasive surgery for the abdominal or the thoracic part?

Which advantages/disadvantages can you describe from your point of view/experience?

|  |
| --- |

**Very distal SCC** (In oesophago-gastric junction)

- Is it allowed to do a trans-hiatal resection with the “wrong histology” if it is located in the oesophago-gastric junction?

|  |
| --- |

**Oesophago-gastric junction carcinoma (Siewert l or ll):**

Indication for transhiatal resection?

|  |
| --- |

Recommended lymph node resection, different for Siewert l versus ll?

|  |
| --- |

**INDUCTION CHEMOTHERAPY**

*There is no evidence for, but a lot of centres give some cycles of chemotherapy before chemoradiation in daily routine.*

Is there a good rationale for induction chemotherapy before chemoradiation? Dependent if definitive CRT or followed by surgery?

|  |
| --- |

Is histology relevant?

|  |
| --- |

Are there reasons against?

|  |
| --- |

How many cycles/weeks are justifiable?

|  |
| --- |

**YOUR SUGGESTIONS**

Any comments?

|  |
| --- |

Any additional questions from your point of view?

|  |
| --- |

Where do you see the most important research field in the next few years? Realistic and unrealistic wishes …

|  |
| --- |
